# Reactive Oxygen Species in Mesenchymal Stem Cell Aging: Implication to Lung Diseases

**DOI:** 10.1155/2015/486263

**Published:** 2015-07-26

**Authors:** Se-Ran Yang, Jeong-Ran Park, Kyung-Sun Kang

**Affiliations:** ^1^Department of Thoracic and Cardiovascular Surgery, School of Medicine, Kangwon National University, Chuncheon 200-701, Republic of Korea; ^2^Institute of Medical Science, Kangwon National University, Chuncheon 200-701, Republic of Korea; ^3^Adult Stem Cell Research Center, College of Veterinary Medicine, Seoul National University, Seoul 151-742, Republic of Korea

## Abstract

MSCs have become an emerging cell source with their immune modulation, high proliferation rate, and differentiation potential; indeed, they have been challenged in clinical trials. Recently, it has shown that ROS play a dual role as both deleterious and beneficial species depending on their concentration in MSCs. Various environmental stresses-induced excessive production of ROS triggers cellular senescence and abnormal differentiation on MSCs. Moreover, MSCs have been suggested to participate in the treatment of ALI/ARDS and COPD as a major cause of high morbidity and mortality. Therapeutic mechanisms of MSCs in the treatment of ARDS/COPD were focused on cell engraftment and paracrine action. However, ROS-mediated therapeutic mechanisms of MSCs still remain largely unknown. Here, we review the key factors associated with cell cycle and chromatin remodeling to accelerate or delay the MSC aging process. In addition, the enhanced ROS production and its associated pathophysiological pathways will be discussed along with the MSC senescence process. Furthermore, the present review highlights how the excessive amount of ROS-mediated oxidative stress might interfere with homeostasis of lungs and residual lung cells in the pathogenesis of ALI/ARDS and COPD.

## 1. Introduction

Human tissue-derived mesenchymal stem cells (MSCs) are emerging as a promising therapeutic approach of cell-based therapy for various diseases including those of neuronal, musculoskeletal, cardiovascular, pulmonary, and autoimmune systems. MSCs can be isolated from a variety of tissues such as bone marrow, adipose tissue, skin dermis, dental pulp, hair follicle, and umbilical cord blood [1–6]. Due to their immunomodulatory and regenerative capacity, MSCs hold great potential; moreover, the advantages regarding no ethical issues unlike embryonic stem cells (ESCs) or induced pluripotent stem cells (iPSCs), a low risk of teratoma formation, and relatively easy obtainment have shown promising results in preclinical and clinical studies. Remarkably, MSCs are responsible for maintaining homeostasis and coordinating tissue repair after tissue injury or inflammation.

The severity of injured organs depends on tissue-specific stem cells, with the capacities for proliferation and differentiation being critical for residual cellular survival and the maintenance of regenerative responses. In almost all tissues, MSCs undergo a replicative senescence “Hayflick limit” after a fixed number of cell divisions. The residual MSCs of aging tissues exhibit a progressive decline, with most biological functions contributing to degenerative changes, and those cells become susceptible to the accumulation of cellular damage and senescence [7]. Recently, it has been shown that the residual MSCs in many tissues are faced with cellular-molecular changes, with age leading to declines in proliferative and functional capacities. Indeed, addressing cell morphology, proliferation, and the maximum number of cell passages are some of the major points to consider in the manufacturing and quality control of human cell therapy medicinal products. Understanding age-related phenomena of MSCs including self-renewal, proliferation, and differentiation capacity is critical for developing cell-based therapeutics for various diseases. Here, we will discuss the roles of ROS in the context of cellular and molecular signaling pathways in MSCs aging.

## 2. ROS, Oxidative Stress, and Cellular Signaling in MSCs Aging

One leading hypothesis, ROS as metabolic side product, may mainly cause the loss of differentiation capacity rather than proliferation of MSCs due to DNA damage accompanied by normal aging. However, MSCs in many tissues are continuously exposed to oxidants endogenously, by intraextracellular metabolism, or exogenously. ROS as a highly unstable species with unpaired electrons include superoxide anion (O_2_^−^) and hydroxyl radical (OH^−^) [8, 9]. ROS is capable of initiating oxidation and causing a variety of cellular responses through the generation of secondary metabolic reactive species. ROS have been shown to be involved in senescence. Moreover, senescent cells are known to have higher levels of ROS than normal cells. Excess ROS is harmful because of its potent ability to interact with a wide range of cellular molecules implicated in cytotoxicity and mutagenic damage. Conversely, a low level of ROS is necessary in order to maintain cell proliferation, self-renewal ability, and regulation of differentiation and serve as intracellular signaling molecules.

**Figure 1:**
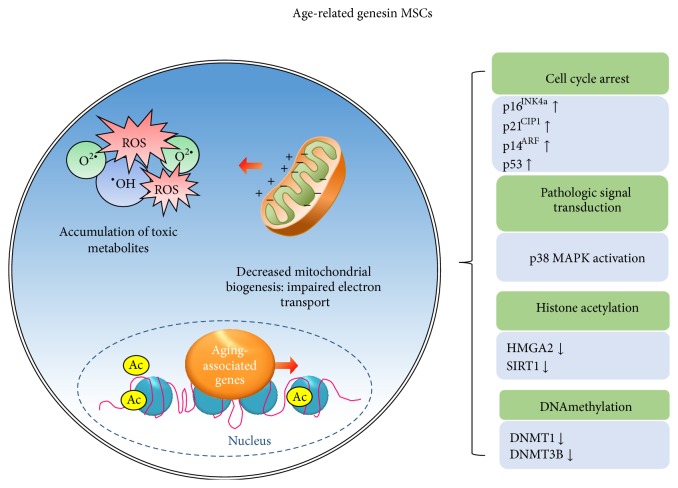
ROS-dependent cellular senescence pathways in MSCs. Similar to other adult somatic cells, MSCs enter replicative senescence after a certain number of cell divisions. ROS are responsible for cellular senescence and cause a direct DNA damage. DNA damage triggers a specific DNA damage response (DDR). DDR activation leads to cell cycle arrest via activation of p53/p21 and/or p16/pRB pathway. In addition, MAPK pathway is required for the acquisition of senescence. P38 plays an important causative role in cellular senescence induced by oxidative stress. Furthermore, ROS regulate major epigenetic processes and can induce DNA methylation and histone acetylation. Understanding the mechanism of senescence of MSCs should provide more effective strategies in transplantation of MSCs into the recipients with age-related diseases inherently associated with increased levels of oxidative stress.

A member of the family of mitogen-activated protein kinases (MAPKs), p38 MAPK, is an important mediator in response to extracellular stressors, such as UV radiation, osmotic shock, hypoxia, and proinflammatory cytokine and oxidative stress, including singlet oxygen, hydrogen peroxide, nitric oxide, and peroxynitrite [10, 11]. Recently, we have shown the effect of replicative senescence on the immunomodulatory ability of MSCs. Aged MSCs in late passage morphologically changed with flattening and enlargement, increasing the SA-*β*-gal activity compared to young MSCs in early passage. Moreover, aged MSCs exhibited a declined immunosuppressive function when CD3/28 and IL-2 were treated to induce the activation and proliferation of T cells [12]. In aged MSCs, proinflammatory stimuli, such as interferon gamma (IFN*γ*) and tumor necrosis factor alpha (TNF*α*), increased the phosphorylation of the p38 MAPK, and this phosphorylation was associated with the downregulation of cyclooxygenase-2(COX2)/prostaglandin E_2_ (PGE_2_), suggesting the immunomodulatory ability of MSCs gradually declines with consecutive passages via the p38 MAPK alteration of COX2/PGE_2_ levels. In agreement with the immunomodulatory function with PGE_2_, MSCs suppress mononuclear cell proliferation and reduce the severity of colitis through the nucleotide-binding oligomerization domain 2 (NOD2) receptor-interacting serine/threonine-protein kinase 2 (RIP2) pathway, leading to the elevation of PGE_2_ production (Figure 1) [13].

As p38 MAPK has participated in molecular interactions during aging, p53 is considered a major mediator in ROS-related signal transduction. It is widely established that p53 as a longevity assurance gene is activated to induce persistently the expression of p21, which may involve the initiation of cell cycle arrest by inhibiting cyclin-dependent kinase (CDK). In interaction with ROS and p53, ROS has been implicated in the phosphorylation of p53 mediated via p38. Structurally, p53 itself is redox-modified due to the presence of cysteine residues containing redox-sensitive thiol groups (–SH). Glutathione is found to interact with either Cys124, 141, or 182 of p53 via a disulfide bond in response to oxidative stress, resulting in the decreased DNA binding activity of p53 [14]. In human endometrium-derived MSCs, hydrogen peroxide with one oxidative stressor treatment induced a rapid phosphorylation of the adaptor protein 53BP1, inducing a DNA damage response (DDR) activation, as well as causing an irreversible arrest of the cell cycle in the G0/G1-phase. In cellular arrest, DDR induced the activation of p53 and upregulated p21 to inactivate pRb. Moreover, the pharmacological inhibition of the p38 MAPK activation abrogated the hydrogen peroxide-induced cell enlargement and flatten morphology, and it is associated with the regulation of mitochondrial ROS production [15]. Although senescent MSCs remain alive, the loss of MSC function with self-renewal and proliferation capacity leads to undergoing apoptosis or senescence. It has been shown that senescent cells upregulate the inhibition of the cell cycle regarding p53/p21 and p16^INK4a^. The cyclin-dependent kinase inhibitor p16^INK4a^ is implicated as a key factor to regulate oxidative stress-induced cell division and arrest the senescence of MSCs and tissue progenitor cells. In endothelial progenitor cells, increased ROS accelerates endothelial progenitor cell senescence by inactivating the PI3K/Akt signaling pathway. In inhibiting PI3K/AKT signaling, the elevated ROS blocks the activation of telomerase [16], and senescent myocardial cells in patients with chronic heart failure exhibited high expressions of p16^INK4a^, as well as telomere shortening [17]. In myocytes, oxidative stress identified by 8-hydroxy-2′-deoxyguanosine increased apoptosis, correlating directly with p16^INK4a^. In addition, increased p16^INK4a^ is associated with the p53 expression and telomere shortening. The process of cardiac stem cells follows a pattern similar to myocytes, indicating this process eventually leads to myocardial regeneration, heart aging, and heart dysfunction [18].

## 3. Chromatin Remodeling during Aging and Age-Related Factors

Recently, the role of p16^INK4a^ has been discussed in the aging of human umbilical cord blood and adipose tissue-derived MSCs. In these MSCs, the p16^INK4a^ expression was increased in senescent MSCs, and the Polycomb group genes and jumonji domain containing three were regulated and governed cellular senescence by the upregulation of p16^INK4a^ [19]. Dynamic chromatin structure changes, including DNA methylation and histone modification, have been demonstrated to be critical in determining stem cell functions. These posttranslational modification-related findings have been applied to address the age-related cellular mechanism in stem cell aging. The process of aging exhibits the profound changes in gene expression profiles and further augmented when MSCs are exposed to DNA damage, which is induced by ROS. In hematopoietic stem cells, the forced increase in intracellular levels of ROS by treatment with a glutathione synthetase inhibitor aggravated the accumulation of DNA damage, resulting in the activation of cell cycle inhibitors, including p16^INK4a^, p14^ARF^, and p21^CIP1^. Moreover, the accumulation of oxidative DNA damage was reproduced in the hematopoietic stem cells of elderly individuals and transplant recipients in the human-to-mouse xenotransplantation study [20]. In a posttranslational modification, histone deacetylases (HDACs) are enzymes that catalyze the removal of acetyl groups in lysine residues of histone tail. In MSCs and tissue-specific stem cells, the downregulation of HDACs has been shown to decrease self-renewal capacity with a loss of stemness, while increasing differentiation markers. Therefore, stem cells undergoing differentiation are somewhat following the features of stem cell aging in the loss of self-renewal and susceptibility to DNA damage. In MSCs, HDAC inhibitors induced aging and spontaneous differentiation into osteogenic lineage with an alteration to histone H3 acetylation and to K9 and K14. The decrease in HDACs was followed by the downregulation of Polycomb group genes, including BMI1, EZH2, and SUZ12, and the upregulation of the jumonji domain containing three was found in senescent MSCs [19]. BMI1 in the Polycomb group genes is known in the maintenance of the self-renewal ability of hematopoietic stem cells by silencing the* INK4a/Arf* locus. It has been shown that BMI1 regulates mitochondrial function by regulating mitochondrial-related genes and ROS generation. Indeed, the cells derived from* Bmi1* knockout mice exhibited impaired mitochondrial function due to the deregulated expressions of genes and led to a significant increase in the intracellular levels of ROS associated with the DNA damage response pathway [21]. In* BMI1*-transgenic mice, hematopoietic stem cells of the overexpression of* Bmi1* retained a better self-renewal capacity and protected against oxidative stress from a culture condition with 20% oxygen. Moreover, buthionine sulfoximine-induced depleted intracellular glutathione and increased endogenous ROS were restored upon the overexpression of* Bmi1 *[22].

In the HDAC class, SIRT1 is a member of the class III HDACs, sharing a catalytic domain of ~275 amino acids with SIRT2–7, and it is a NAD-dependent protein to mediate the deacetylation of histone and nonhistone proteins in moderating lifespan extension [23]. Though SIRT1 acts as a growth suppressor gene of telomerase-immortalized cells, the expression of SIRT1 was gradually decreased with the serial cell passage [24]. In the knockdown of SIRT1 of MSCs, cellular senescence was accelerated with the accumulation of the protein p16, whereas the overexpression of SIRT1 delayed senescence [25]. The relationship between SIRT1 and ROS has been demonstrated regarding the aspect of mitochondrial biogenesis in that a cause of aging is the oxidation of macromolecules through the generation of the ROS of mitochondria [26]. Briefly, mitochondrial biogenesis activates the SIRT1 expression, and its upregulation of SIRT1 induces an increase in the size of mitochondria, leading to the mitigation of hyperpolarization. Thus, the generation of ROS in mitochondria becomes reduced due to the stalling of electrons in the electron transport chain. Therefore, calorie restriction has been first introduced to extend life span by slowing carbohydrate use, leading to reductions in the production of ROS. Furthermore, calorie restriction is associated with increased SIRT1-mediated PGC-1*α* deacetylation at several lysine residues, and this increase during calorie restriction resulted in mitochondrial biogenesis in the muscle and white fat of mice [27].

In chromatin remodeling, high mobility group A (HMGA2) as a nonhistone chromatin-binding protein family includes its isoforms HMGA1 and HMGA2. These chromatin-associated proteins lack their own intrinsic transcriptional activity, instead of binding to AT-rich DNA sequences and affecting related transcription factors by altering the chromatin structure [28]. HMGA2 has been associated with neoplasia with diverse oncogenic effects on the cell cycle by inducing cyclin A and the p53-mediated apoptotic pathway [29]. Recently, the role of HMGA2 as a developmental regulator has been featured in the self-renewal of stem cells. HMGA2 is highly expressed in undifferentiated cells during embryogenesis; however, its expression gradually declined along with fetal development progress [30]. In mouse neural stem cells, HMGA2 was specifically increased but declined with age through the regulation of p16^INK4a^ and p19^ARF^ [31]. Despite HMGA2 not being required for the self-renewal of neural stem cells of old mice, the role of HMGA2 is emerging in the maintenance of MSCs with age. In human umbilical cord blood-derived-MSCs, the overexpression of HMGA2 reduced the SA-*β*-gal activity and enhanced the proliferation rate with a remarkable change in gene profiles. In addition, the overexpression of HMGA2 was associated with the upregulation of the PI3K/Akt/mTOR/p70S6K cascade and suppressed the expressions of p16^INK4A^ and p21^CIP1/WAF1^ [32]. Currently, it is now known whether ROS mediated induction of HMGA2 may lead aging of MSCs. However, in various cancers, a high level of HMGA2 has been detected, and it is believed that HMGA2-related ROS production modifies redox regulation, leading to mold cellular phenotypes. In the epithelial-mesenchymal transition, TGF-*β* sustained increases in the generation of ROS, as well as the induction of HMGA2 with a decrease in mitochondrial membrane potential and the glutathione level. When exogenous mitochondrial thioredoxin was treated in EMT, the TGF-*β* mediated induction of HMGA2 was impaired in mammary epithelial cells [33]. These findings regarding the underlying mechanism in epithelial-mesenchymal transition might be extended to delay stem cell aging in response to ROS.

DNA methylation is involved in diverse biological process with the addition of a methyl group to the CpG dinucleotide in DNA. This methylation pattern is regarded as a fundamental constituent of the molecular mechanism, as well as a parameter to understand the correlation with chromatin structure remodeling. The alteration of the DNA methylation landscape has been analyzed to determine the characteristics between age-associated hematopoietic stem cell decline and the epigenome based on a genome-wide DNA methylation analysis. In hematopoietic stem cells, the specific DNA hypermethylation of Polycomb repressive complex 2 was accompanied by enforced proliferation-dependent aging [34]. Recently, it has been demonstrated that DNA methyltransferase enzymes (DNMTs) regulate histone marks, transcriptional enzymes, and the CpG island methylation status during the replicative senescence of MSCs. In MSCs, the inhibition of DNMT 1 and 3B with 5-azacytidine increased SA-*β*-gal activity through increased levels of p16^INK4A^ and p21^WAF/Cip1^ as a result of DNMTs downregulating the CDK2 and CDK4 expressions in the G0/G1 phase of the cell cycle. In addition, the specific inhibition of DNMT1 and DNMT3b with siRNAs decreased EZH2 and BMI1, which are regulatory mediators of Polycomb repressive complexes at the mRNA and protein levels by controlling the expressions of microRNA-200c and 214 on the genomic region [35]. The ROS-mediated mechanism with DNMTs and Polycomb repressive complex genes has been described in cancer cells. Hydrogen peroxide induced the large complex of DNMT1 and 3B containing Sirtuin1 and Polycomb repressive complex 4. In these complexes, SIRT1 interacted with DNMT1 and they were recruited to hydrogen peroxide-induced double strand breaks [36]. In murine melanocytes, superoxide anion (O_2_^−^), a species of ROS, upregulated the expressions of DNMTs through the RAS signaling pathway, which is capable of activating the DNMT promoter [37]. In neural stem cells, Bose et al. have shown dexamethasone-induced changes in global DNA methylation accompanied by decreased expressions of DNMTs (Figure 1). This phenomenon was underlying the transcriptional repression of the mitochondrial-respiratory chain enzymes of complex 1 (*Nd3*) and complex III (*Cytb*), leading to a long-lasting increased susceptibility to oxidative stress, such as the higher generated levels of intracellular ROS [38].

## 4. The Roles of ROS in Residual Stem Cells of Lung Tissue

Lungs are continuously exposed to exogenous environmental pollutants in the ambient air, for example, cigarette smoke, dust, and ozone, and systemically to ROS generated from xenobiotic compounds. In the lung, the primary function is to facilitate the diffusion of gases in the exchange of carbon dioxide for oxygen (O_2_) across the alveoli and capillaries. Adult human lungs exchange between 10,000 to 20,000 liters of air daily, and this volume includes toxic particles presenting in the work environment, as well as in the inhalation of particles in polluted air by the general population [39]. In human lungs, endogenous lung stem cells and progenitor cells are known as regenerative populations essential for cellular maintenance and injury repair, and those populations are considered facultative progenitor cells: basal, Clara-like, Clara, pulmonary neuroendocrine, and type II alveolar epithelial cells [40]. Yin and colleagues have reported that aged mice sustained extensive losses of alveolar types I and II cells and delayed the regeneration of alveolar type II cells and their precursors (prosurfactant protein C-positive bronchiolar epithelial cells) compared with young mice. Moreover, the aged mice were more susceptible to influenza-induced morbidity and mortality, which has been associated with impaired immunity [41]. In mouse airway basal stem cells, low levels of ROS increased their ROS in culture and exhibited a higher proliferative capacity compared to high levels of ROS of mouse airway basal stem cells. The low or moderate levels of ROS were associated with the proliferation of airway basal stem cells via a correlation with the G1/M transition during the cell cycle. To determine the cellular mechanism in redox regulation, Nrf2 was targeted because Nrf2 and its repressor protein Keap1 are regarded as a major interaction to balance intracellular redox levels. In* Nrf2* ^−/−^ mice, Nrf2 deficiency decreased the proliferation of airway basal stem cells with a lack of sphere formation, and Notch1-mediated proliferation was governed by Nrf2 in ROS-induced self-renewal for repair after injury [42]. Recently, lung mesenchymal stem cells have been isolated from nasal mucosa and lung compartments [43]. The isolated cells expressed cell-surface proteins, CD73, CD105, CD166, and CD90, commonly found on MSCs. Lama et al. reported that the isolated fibroblast-like cells from the lower respiratory tract of human lung transplant recipients have shown multiple connective tissue lineages including osteocytic, adipocytic, and chondrocytic differentiation [44]. Although there is a growing number of reports of putative MSC-like cells from lung tissues, the potential physiologic or pathophysiologic role of these cell remains unknown.

## 5. Chronic Obstructive Pulmonary Disease (COPD)

**Figure 2:**
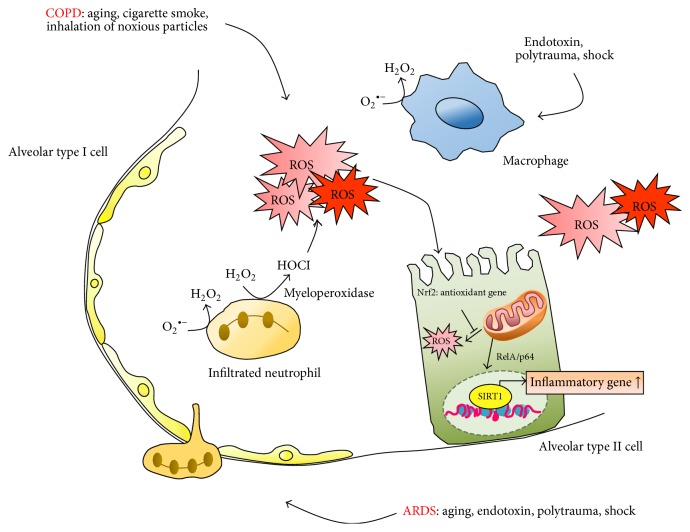
The role of ROS in COPD and ARDS. In COPD, aging and cigarette smoke increase the generation of ROS, leading to the upregulation of inflammatory signaling in alveolar type II cells. In ARDS, endotoxin and shock induce the infiltration of neutrophils into interstitial, and chromatin changes are involved. In the pathogenesis of COPD and ARDS, ROS-induced neutrophils are recruited in alveoli, and production of ROS is rapidly accumulated, derived from infiltrated neutrophils, and activated macrophages.

The prevalence of patients with chronic obstructive pulmonary disease (COPD) is two to three times higher in individuals over age 60. In 2020, COPD will be the third leading cause of death worldwide according to the global burden of disease study [45]. This increased economic burden of COPD in the elderly population is suggested to be due to age-associated structural and functional changes in the lung, leading to an increase in the pathogenetic susceptibility to COPD. Generally, premature replicative senescence resulting in telomere shortening and cigarette smoke-induced premature stress-related senescence are two major forms in aging and COPD. Cigarette smoking is believed to be the greatest risk factor for developing COPD in genetically susceptible individuals. The classical definition of COPD is an airway and lung inflammation, mucociliary dysfunction, alveolar destruction, and airway fibrosis in the response of the lungs to the inhalation of noxious particles or toxic gases [46]. COPD has been described as accelerating lung aging, and the role of ROS is emerging to regulate aging-associated inflammation and structural changes in the lungs. Accelerated cellular senescence resulting from cigarette smoke exposure induced mitochondrial fragmentation and increased mitochondrial ROS production in COPD lung tissues [47]. As mentioned above, it has been demonstrated that SIRT1 deacetylase levels are reduced in chronic inflammatory conditions and aging where ROS is induced. In* SIRT1* ^+/−^ mice, a spontaneous airspace enlargement and age-dependent reductions in SIRT1 levels were shown, and cigarette smoke exposure rapidly augmented airspace enlargement via the FOXO3-mediated pathway on cellular senescence and emphysematous changes [48]. Similarly, in the lungs of patients with COPD, the SIRT1 level was decreased, and the decreased SIRT1 activity induced a proinflammatory cytokine IL-8 release. In addition, in human monocyte-macrophage cells, cigarette smoke extract caused posttranslational modifications of SIRT1 by 4-hydroxynonenal and 3-nitrotyrosine, and this modification was associated with increased acetylation and the activation of RelA/p65 NF-*κ*B (Figure 2) [49]. Mesenchymal cells (fibroblasts and endothelial cells) and their progenitors in COPD are mainly demonstrable, correlating to replicative senescence in alveolar parenchyma.

## 6. Acute Lung Injury/Acute Respiratory Distress Syndrome (ARDS)

Acute lung injury [16] and its most severe form, acute respiratory distress syndrome (ARDS), are frequent complications and are responsible for a significant mortality rate of 50–80% [50]. ALI/ARDS can result from clinical conditions including polytrauma, hemorrhagic shock, and severe burns. However, ARDS is almost invariably associated with sepsis through lipopolysaccharide-mediated action. Currently, patients with ARDS receive mechanical ventilation with positive end-expiratory pressure and high O_2_ concentrations. These therapeutic supports have been shown to maintain oxygen concentration; however, they may exacerbate the primary injury. In ARDS, there are many potential sources of ROS from infiltrated neutrophils and inhaled gases with high concentrations of oxygen. In patients with ARDS, the levels of hydrogen peroxide and 4-hydroxynonenal were increased, whereas antioxidant enzymes including superoxide dismutase and glutathione were dropped with increased levels of ROS [51]. The activation of neutrophils from pulmonary circulation causes the release of ROS to be able to disrupt the vascular endothelium layer, leading to an infiltration of the pulmonary interstitial. Neutrophils have been known to produce ROS and contain NADPH oxidase, generating a huge amount of O_2_^−^, which is responsible for a respiratory burst. In addition, the release of myeloperoxidase from neutrophil catalyzes the production of hypochlorous acid (HOCl) from hydrogen peroxide [52]. HOCl, a potent oxidant formed from hydrogen peroxide by released myeloperoxidase, is increasingly considered as an ROS-related major risk factor for ARDS (Figure 2). Accumulating data suggest the respiratory function, and the ratio of arterial PO_2_ to inspired O_2_ fraction in patients with ARDS over age 60 was significantly less compared with the young [53]. In juvenile mice at 21 d, inflammatory responses were less susceptible to mechanical ventilation, showing that the injury responses are acquired with age as a result of coordinated changes in gene expression, apoptotic, and TGF-*β* pathways [54]. In the differentiation of mouse MSCs into alveolar type II cells, Wnt5a was protected against oxidative stress-mediated cellular toxicity, and this pathway promoted the migration of mouse MSCs [55]. Alveolar type II cells are considered critical for the repair of injured lung tissues and homeostasis [56]. Although alveolar type II cells have been described as lung progenitors for repair, it is unclear how those cells contribute to repairing the pulmonary epithelium in ALI/ARDS.

## 7. Therapy Approaches of MSCs for ARDS and COPD

MSCs can act provided novel insight their potential applicability for clinical use in the treatment of lung disease including COPD and ARDS. Several studies evaluated therapeutic effect of MSCs through validation of the immunomodulatory and anti-inflammatory ability of MSCs (Table 1). MSCs are able to migrate to sites of tissue injury and have strong immunosuppressive properties that can be exploited for successful autologous as well as heterogonous transplantations [57]. In a mouse model of LPS-induced acute lung injury, MSCs administration into the lung triggers downregulation of proinflammatory factors such as TNF-*α* and MIP-2 in the bronchoalveolar lavage fluid (BALF) and plasma while increasing the anti-inflammatory cytokine IL-10 [58]. Similarly, in a rat model of LPS-induced lung injury, injection of human umbilical cord-derived MSCs not only increased the survival rate of rats suffering from LPS-induced lung injuries but also significantly reduced systemic and pulmonary inflammation such as reduced lung edema, lung wet/dry ratio, protein concentration, neutrophil counts, and MPO activity in BALF [59]. Also, in* E. coli*-induced acute lung injury in mice, intratracheal administration of human MSCs leads to improved survival of mouse and attenuated lung injury mainly due to suppression of proinflammatory cytokines (IL-1*α*, IL-1*β*, IL-6, TNF-*α*, and MIP-2) and MPO activity as well as reducing the elevated lung water content [60]. Taken together, the MSCs have been used extensively in different mammalian models for lung injury/ARDS and led to a significant decrease in pathophysiological features of lung inflammation (edema, protein leakage, neutrophil infiltration, hemorrhage, and intra-alveolar thickening) with attenuation of proinflammatory cytokines.

COPD comprises two major phenotypes which are chronic bronchitis and emphysema. Interestingly, it has been demonstrated that mouse MSCs are able to ameliorate the emphysematous changes and reduce destruction in elastase-induced emphysema model through upregulation of hepatocyte growth factor (HGF), epithelial growth factor (EGF), and secretory leukocyte protease inhibitor (SLPI) in the lung [75]. Moreover, administration of rat MSCs improves emphysema and destructive function induced by CS exposure via decrease of proinflammatory mediators (TNF-*α*, IL-1*β*, MCP-1, and IL-6) and protease (MMP9 and MMP12) and increase of vascular endothelial growth factor (VEGF), VEGF receptor 2, and transforming growth factor (TGF-*β*) and consequently reduced pulmonary cell apoptosis [73]. In the cigarette smoke-induced emphysema model of COPD, MSCs administration and treatment of conditioned media of MSC reduced pulmonary cell apoptosis, attenuated the mean pulmonary arterial pressure, and inhibited muscularization in small pulmonary vessels [71]. In addition, another study showed that the protective effect of MSC transplantation on the rat model of papain-induced pulmonary emphysema may be partly mediated by upregulating VEGF-A expression and inhibiting the apoptosis of lung cells [70]. Therefore, MSCs-based therapies may represent new therapeutic approaches for COPD that currently lacks efficient treatment.

## 8. Conclusion

MSCs aging is proposed to potentially contribute to organismal aging leading to loss of tissue homeostasis. While high level of ROS is generally accepted to promote MSC aging through the induction of oxidative stress, physiological levels of ROS may rather improve proliferation and differentiation capacity. It is essential to consider the role of ROS in the aging process of MSCs since MSCs and their regenerative potential have revealed a critical therapeutic approach against aging. In lung diseases, the excessive amount of ROS from the ambient air might be associated with respiratory failure through unsuspected mechanisms that regulate declines in the residual stem cell function with age. Much work remains to be done to understand the ROS-mediated mechanisms that regulate residual stem cells. Elucidating these mechanisms will be critical to understanding how stem cell based or antioxidant therapies are effective in certain tissues including lungs.

## Figures and Tables

**Table 1:** Potential mechanisms of action of mesenchymal stem cell in animal models of lung diseases.

Source	Injury model	Cell delivery route	Finding and mechanism of action	Reference
Acute respiratory distress syndrome (ARDS)/acute lung injury/pneumonia				

Bone marrow-derived MSCs	Murine model in LPS-induced ALI (i.t)	i.t mBM-MSCs 5 × 10^5^ cells 1 h/4 h/24 h after injury	Both functional and survival advantages with histological improvement in the severity of lung injury without engrafting through to stem cell chemoattractants	[58]
	Rodent model in endotoxin-induced ALI (i.v)	i.v rBM-MSCs 7.1 × 10^6^ cells 2 h after injury	The beneficial effect of MSCs overexpressing HO-1 might be achieved through the engraftment of differentiated MSCs in lung through secretion of paracrine factors	[61]
	Rodent model in paraquat poisoning-induced ALI (i.p)	i.v rBM-MSCs 1 × 10^6^ cells 6 h after injury	Inhibit the release of inflammatory mediator, lung edema, and lipid peroxidation	[62]
	Rodent model in LPS-induced ALI (i.t)	Intrapleural delivery rBM-MSCs 1 × 10^6^ cells immediately after injury	Attenuate the severity of ALI by mediating paracrine/endocrine repair mechanism than by the cell engraftment mechanism	[63]
	Murine model in LPS-induced ALI (i.t)	i.t mBM-MSC MVs 12 h after injury	The therapeutic properties of MSCs can be recapitulated by the MV that MSCs actively secrete in culture through KGF	[64]
	Murine model in LPS- or CLP-induced ALI (i.p)	i.t hBM-MSCs 2 × 10^6^ cells 24 h after injury	MSCs therapy at day 1 reduces lung inflammation and remodeling for each type of initial insult triggering extrapulmonary ARDS; MSCs increase MMP8 and decreaseTIMP1; MSCs shift macrophage	[65]

Adipose tissue-derived MSCs	Rodent model in IR-induced ALI	i.v mASCs 4.8 × 10^6^ cells 1 h and 6 h after injury	Autologous ASCs suppress inflammatory response and oxidative stress (increased NAD(P)H, HO-1) as well as enhancement of angiogenesis (VCAM1, ICAM-1)	[66]
	Rodent model in LPS-induced ALI (i.v)	i.v hASCs 2 × 10^6^ cells 30 min after injury	Decrease inflammatory cytokine levels in serum and lung as well as reduce alveolar inflammatory cell infiltration in the lung and protected multiorgan injury	[67]
	Murine model in LPS-induced ALI (i.t)	O.A mASCs or hASCs 7.5 × 10^5^ cells 4 h after injury	Attenuates neutrophil influx and inflammation due to the increased production of IL-10	[68]

Umbilical cord-derived MSCs	Murine model in LPS-induced ALI (i.t)	i.t hUC-MSCs 1 × 10^6^ cells 3-4 h after injury	Several clinical advantages that provide expansion of CD4+CD25+Foxp3+Treg cells, balancing anti- and proinflammatory factors as well as bacterial clearance	[60]
	Rodent model in LPS-induced ALI (i.t)	i.v hUC-MSCs 5 × 10^5^ cells 1 h after injury	Reduces TNF-*α*, IL-1*β*, and IL-6 but not IL-10 as well as oxidative stress	[59]

MSCs from other tissues	Murine model in LPS-induced ALI (i.t)	i.v human orbital fat-derived MSCs 3 × 10^5^ cells 20 min after injury	Systemic orbital fat-derived stem/stromal cells are effective in modulating inflammation	[69]

Chronic obstructive pulmonary disease (COPD)/emphysema				

Bone marrow-derived MSCs	Rodent model in CS-induced emphysema (6 m)	i.t or i.v rBM-MSCs 6 × 10^5-6^ cells and rBMMSC-CM 0.3 mL 5 w after injury	Increased VEGF-A and inhibited the apoptosis (Bax, Bcl-2) of lung alveolar cells; TNF-*α*-mediated VEGF-A secretion by VEGF The effectiveness of MSC-CM was similar to that of BMCs and MSCs, supporting a paracrine mechanism as well as decreasing apoptosis May recover lung fibroblast from CS-induced damage through inhibition of caspase-3, induction of proliferation, and restoration of lung fibroblast repair function via PI3K/Akt pathway	[70–72]
	Rodent model in CS-induced emphysema (11 w)	i.t rBM-MSCs or hBM-MSCs 6 × 10^6^ cells after injury	A therapeutic potential in parenchymal repair by increased levels of growth factors and decreased cell apoptosis through VEGF, VEGF receptor, and TGF*β*-1 Relieve airway inflammation through inhibition of COX-2/PGE_2_ in alveolar macrophages, mediated by the p38 MAPK and ERK pathway	[73, 74]

Adipose tissue-derived MSCs	Murine model in PPE-induced emphysema (i.t)	i.t mASCs 5 × 10^5^ cells 2 w after injury	ASCs ameliorate damage of alveolar structure through the release of soluble humoral factor (HGF, EGF, and SLP1)	[75]

(i.t): intratracheal; (i.v): in vein; (i.p): intraperitoneal.
